# Supplementary material for: Population-Based External Validation of the EASIX Scores to Predict CAR T-Cell-Related Toxicities
Source: Cancers (Basel). 2023 Nov 16;15(22):5443. doi: 10.3390/cancers15225443 (PMC10670876; doi:10.3390/cancers15225443)
Supplement: Supplementary file 1 [file cancers-15-05443-s001.zip › cancers-2664943-supplementary.pdf]

Table S1. Summary statistics of EASIX, m-EASIX and s-EASIX scores at baseline, pre-LD and infusion grouped for grade < 2 and grade ≥ 2.

			CRS		ICANS		Overall
			Grade < 2	Grade ≥ 2	Grade < 2	Grade ≥ 2	
Baseline	EASIX	Median (IQR)	1.30 (0.80–2.37)	1.59 (0.89–2.73)	1.19 (0.78–2.34)	1.61 (0.92–2.74)	1.31 (0.81–2.60)
		Range	(0.29–145.40)	(0.36–42.00)	(0.36–42.00)	(0.29–145.40)	(0.29–145.40)
		N	76	61	82	55	137
	m-EASIX	Median (IQR)	2.32 (0.68–5.75)	4.33 (0.83–9.76)	3.22 (0.86–8.06)	2.08 (0.54–9.56)	2.61 (0.68–8.79)
		Range	(0.02–76.51)	(0.04–106.63)	(0.02–41.25)	(0.06–106.63)	(0.02–106.63)
		N	56	44	56	44	100
	s-EASIX	Median (IQR)	1.35 (0.99–2.44)	1.42 (1.09–2.90)	1.34 (0.95–2.44)	1.63 (1.08–3.23)	1.39 (1.01–2.62)
		Range	(0.36–98.11)	(0.58–45.83)	(0.47–45.83)	(0.36–98.11)	(0.36–98.11)
		N	77	61	82	56	138
Pre-LD	EASIX	Median (IQR)	1.13 (0.79–2.18)	1.45 (0.82–2.51)	1.06 (0.77–1.68)	1.72 (0.98–3.01)	1.29 (0.82–2.27)
		Range	(0.32–23.37)	(0.28–62.75)	(0.28–62.75)	(0.32–27.35)	(0.28–62.75)
		N	84	68	89	63	152
	m-EASIX	Median (IQR)	0.92 (0.30–3.28)	1.67 (0.44–13.25)	0.93 (0.27–4.22)	2.37 (0.54–10.61)	1.27 (0.35–6.46)
		Range	(0.03–211.72)	(0.04–383.22)	(0.03–383.22)	(0.05–211.72)	(0.03–383.22)
		N	83	58	82	59	141
	s-EASIX	Median (IQR)	1.36 (0.92–2.19)	1.52 (0.94–3.36)	1.27 (0.91–1.78)	1.81 (1.03–3.71)	1.40 (0.92–2.61)
		Range	(0.34–17.07)	(0.33–69.33)	(0.33–69.33)	(0.34–26.87)	(0.33–69.33)
		N	84	68	89	63	152
Infusion	EASIX	Median (IQR)	1.39 (0.89–2.12)	1.82 (0.95–4.40)	1.32 (0.84–2.20)	1.83 (0.97–4.56)	1.44 (0.93–3.08)
		Range	(0.04–16.58)	(0.17–36.44)	(0.04–36.44)	(0.39–23.02)	(0.04–36.44)
		N	71	52	72	51	123
	m-EASIX	Median (IQR)	3.40 (0.76–8.39)	6.73 (2.24–23.53)	3.56 (0.75–8.41)	6.73 (2.21–22.90)	4.64 (1.07–12.97)
		Range	(0.08–247.18)	(0.04–351.06)	(0.08–351.06)	(0.04–247.18)	(0.04–351.06)
		N	66	49	69	46	115
	s-EASIX	Median (IQR)	1.78 (1.23–2.67)	2.24 (1.35–4.99)	1.66 (1.22–3.08)	2.23 (1.44–5.01)	1.95 (1.26–3.52)
		Range	(0.49–27.15)	(0.27–50.33)	(0.27–50.33)	(0.49–29.07)	(0.27–50.33)
		N	71	52	72	51	123

CRS = cytokine release syndrome; ICANS = immune effector cell-associated neurotoxicity syndrome; IQR= interquartile range; EASIX = Endothelial Activation and Stress Index; m-EASIX = modified EASIX score; s-EASIX = simplified EASIX score; pre-LD = pre-lymphodepleting chemotherapy (day-15 until day -5).

Table S2. Summary statistics of EASIX, m-EASIX and s-EASIX scores at baseline, pre-LD and infusion grouped for grade < 3 and grade ≥ 3.

			CRS		ICANS		CRS or ICANS	
			Grade < 3	Grade ≥ 3	Grade < 3	Grade ≥ 3	Grade < 3	Grade ≥ 3
Baseline	EASIX	Median (IQR)	1.30 (0.81–2.55)	2.68 (1.24–2.72)	1.25 (0.80–2.53)	1.64 (1.12–2.68)	1.23 (0.81–2.47)	1.68 (1.00–2.70)
		Range	(0.29–145.40)	(0.70–3.78)	(0.29–145.40)	(0.46–18.74)	(0.29–145.40)	(0.46–18.74)
		N	130	7	108	29	103	34
	m-EASIX	Median (IQR)	2.79 (0.70–8.80)	1.24 (0.47–4.94)	2.97 (0.70–8.80)	1.85 (0.55–7.32)	2.99 (0.89–8.81)	1.76 (0.52–7.26)
		Range	(0.02–106.63)	(0.07–26.13)	(0.02–76.51)	(0.06–106.63)	(0.02–76.51)	(0.06–106.63)
		N	94	6	78	22	73	27
	s-EASIX	Median (IQR)	1.37 (0.97–2.60)	2.52 (1.32–3.33)	1.36 (0.93–2.59)	1.63 (1.11–3.01)	1.35 (0.89–2.59)	1.55 (1.16–3.01)
		Range	(0.36–98.11)	(1.18–4.51)	(0.36–98.11)	(0.61–15.20)	(0.36–98.11)	(0.61–15.20)
		N	131	7	108	30	103	35
Pre-LD	EASIX	Median (IQR)	1.29 (0.82–2.26)	1.30 (0.82–3.58)	1.13 (0.79–2.21)	1.46 (1.00–2.65)	1.13 (0.80–2.22)	1.45 (0.87–2.61)
		Range	(0.28–62.75)	(0.56–5.62)	(0.28–62.75)	(0.32–23.37)	(0.28–62.75)	(0.32–23.37)
		N	144	8	120	32	114	38
	m-EASIX	Median (IQR)	1.28 (0.35–6.55)	0.91 (0.60–1.63)	1.14 (0.33–6.24)	2.16 (0.54–6.72)	1.22 (0.34–6.55)	1.58 (0.54–5.72)
		Range	(0.03–383.22)	(0.08–61.53)	(0.03–383.22)	(0.05–211.72)	(0.03–383.22)	(0.05–211.72)
		N	134	7	111	30	106	35
	s-EASIX	Median (IQR)	1.40 (0.92–2.59)	1.35 (0.93–4.25)	1.37 (0.92–2.35)	1.77 (1.02–2.76)	1.38 (0.92–2.49)	1.64 (0.96–2.69)
		Range	(0.33–69.33)	(0.85–5.70)	(0.33–69.33)	(0.51–17.07)	(0.33–69.33)	(0.51–17.07)
		N	144	8	120	32	114	38
Infusion	EASIX	Median (IQR)	1.43 (0.93–2.81)	2.56 (1.12–6.15)	1.38 (0.83–2.46)	2.11 (0.99–4.35)	1.39 (0.84–2.37)	2.10 (0.99–4.29)
		Range	(0.04–36.44)	(0.66–23.02)	(0.04–36.44)	(0.39–23.02)	(0.04–36.44)	(0.39–23.02)
		N	118	5	98	25	95	28
	m-EASIX	Median (IQR)	4.64 (0.96–12.97)	7.99 (3.27–69.38)	4.45 (0.77–12.00)	5.84 (1.67–26.61)	4.55 (0.76–12.27)	5.45 (1.47–20.40)
		Range	(0.04–351.06)	(1.13–241.54)	(0.04–351.06)	(0.06–241.54)	(0.04–351.06)	(0.06–241.54)
		N	111	4	93	22	90	25
	s-EASIX	Median (IQR)	1.94 (1.26–3.32)	2.26 (1.36–10.08)	1.81 (1.22–3.12)	2.49 (1.56–4.97)	1.81 (1.22–3.18)	2.30 (1.51–4.82)
		Range	(0.27–50.33)	(1.02–29.07)	(0.27–50.33)	(0.72–29.07)	(0.27–50.33)	(0.72–29.07)
		N	118	5	98	25	95	28

CRS = cytokine release syndrome; ICANS = immune effector cell-associated neurotoxicity syndrome; IQR= interquartile range; EASIX = Endothelial Activation and Stress Index; m-EASIX = modified EASIX score; s-EASIX = simplified EASIX score; pre-LD = pre-lymphodepleting chemotherapy (day-15 until day -5).

Table S3. Analysis of the association between the EASIX score and its derivates and CRS and ICANS

		CRS grade ≥ 2			ICANS grade ≥ 2			CRS grade ≥ 3			ICANS grade ≥ 3			CRS/ICANS grade ≥ 3		
		OR	95% CI	*p*	OR	95% CI	*p*	OR	95% CI	*p*	OR	95% CI	*p*	OR	95% CI	*p*
EASIX*	Baseline	1.08	0.85 - 1.37	0.52	1.20	0.94 - 1.54	0.15	1.13	0.68 - 1.87	0.64	1.02	0.77 - 1.37	0.87	1.02	0.78 - 1.34	0.89
	Pre-LD	1.19	0.93 - 1.52	0.17	1.31	1.02 - 1.68	**0.04**	1.07	0.64 - 1.79	0.81	1.12	0.84 - 1.48	0.45	1.05	0.80 - 1.38	0.71
	Infusion	1.17	0.93 - 1.47	0.18	1.28	1.00 - 1.62	**0.05**	1.32	0.83 - 2.10	0.23	1.23	0.93 - 1.61	0.14	1.18	0.92 - 1.53	0.20
m-EASIX*	Baseline	1.04	0.91 - 1.19	0.59	1.03	0.90 - 1.18	0.64	0.91	0.68 - 1.23	0.54	0.93	0.78 - 1.11	0.44	0.92	0.78 - 1.08	0.29
	Pre-LD	1.11	0.99 - 1.24	0.08	1.11	0.99 - 1.25	0.06	0.96	0.75 - 1.23	0.75	1.04	0.91 - 1.18	0.59	1.00	0.88 - 1.13	0.99
	Infusion	1.10	0.97 - 1.24	0.14	1.10	0.97 - 1.25	0.13	1.16	0.85 - 1.58	0.35	1.05	0.90 - 1.22	0.53	1.04	0.90 - 1.20	0.62
s-EASIX*	Baseline	1.13	0.87 - 1.48	0.35	1.29	0.98 - 1.70	0.07	1.22	0.73 - 2.02	0.45	0.99	0.72 - 1.37	0.96	1.00	0.74 - 1.35	0.99
	Pre-LD	1.22	0.94 - 1.57	0.14	1.33	1.02 - 1.73	**0.04**	1.08	0.63 - 1.85	0.77	1.09	0.81 - 1.47	0.58	1.02	0.77 - 1.37	0.87
	Infusion	1.20	0.93 - 1.54	0.16	1.28	0.99 - 1.67	0.06	1.34	0.82 - 2.18	0.24	1.22	0.91 - 1.64	0.19	1.16	0.87 - 1.54	0.30

* Log2 transformed. EASIX = Endothelial Activation and Stress Index; m-EASIX = modified EASIX score; s-EASIX = simplified EASIX score; CI = confidence interval; CRS = cytokine release syndrome; ICANS = immune effector cell-associated neurotoxicity syndrome; LDH = lactate dehydrogenase; pre-LD = pre-lymphodepleting chemotherapy (day-15 until day -5);OR = odds ratio; *p* = *p*-value; ULN = upper limit of normal. Bold indicates statistically significant values.

Table S4. Analysis of the association of laboratory parameters and CRS and ICANS.

		CRS grade ≥ 2			ICANS grade ≥ 2			CRS grade ≥ 3			ICANS grade ≥ 3			CRS/ICANS grade ≥ 3		
		OR	95% CI	*p*	OR	95% CI	*p*	OR	95% CI	*p*	OR	95% CI	*p*	OR	95% CI	*p*
Platelets*	Baseline	1.04	0.74 - 1.44	0.83	0.63	0.44 - 0.91	**0.02**	1.28	0.54 - 3.03	0.57	0.85	0.58 - 1.24	0.40	0.93	0.64 - 1.34	0.68
	Pre-LD	0.99	0.73 - 1.36	0.97	0.74	0.52 - 1.03	0.08	1.22	0.54 - 2.80	0.63	0.91	0.63 - 1.31	0.60	0.95	0.67 - 1.36	0.80
	Infusion	0.96	0.71 - 1.30	0.79	0.73	0.53 - 1.00	**0.05**	0.84	0.45 - 1.57	0.58	0.82	0.57 - 1.16	0.26	0.82	0.58 - 1.15	0.24
Creatinine*	Baseline	0.80	0.39 - 1.62	0.53	0.79	0.38 - 1.63	0.52	0.58	0.09 - 3.66	0.55	1.30	0.56 - 3.02	0.54	1.17	0.52 - 2.63	0.70
	Pre-LD	0.94	0.41 - 2.12	0.87	1.11	0.49 - 2.54	0.80	0.90	0.14 - 5.69	0.91	1.49	0.55 - 4.01	0.43	1.41	0.55 - 3.59	0.47
	Infusion	1.09	0.59 - 2.01	0.79	1.36	0.68 - 2.70	0.38	1.35	0.27 - 6.70	0.71	1.42	0.58 - 3.45	0.44	1.56	0.66 - 3.68	0.31
CRP*	Baseline	1.02	0.86 - 1.20	0.84	0.98	0.83 - 1.15	0.78	0.84	0.58 - 1.20	0.32	0.91	0.74 - 1.13	0.40	0.89	0.73 - 1.08	0.23
	Pre-LD	1.11	0.96 - 1.27	0.16	1.09	0.95 - 1.25	0.24	0.92	0.68 - 1.25	0.59	1.03	0.88 - 1.21	0.71	1.00	0.85 - 1.16	0.95
	Infusion	1.09	0.93 - 1.28	0.29	1.07	0.91 - 1.26	0.42	1.13	0.72 - 1.80	0.58	1.00	0.82 - 1.21	0.99	1.00	0.83 - 1.21	0.98
LDH*	Baseline	1.44	0.94 - 2.20	0.09	0.95	0.62 - 1.45	0.80	1.99	0.91 - 4.34	0.08	0.73	0.42 - 1.29	0.28	0.89	0.54 - 1.44	0.62
	Pre-LD	2.00	1.19 - 3.35	**0.01**	1.36	0.85 - 2.18	0.20	1.70	0.73 - 3.99	0.22	1.08	0.61 - 1.89	0.80	0.98	0.57 - 1.68	0.94
	Infusion	1.63	1.00 - 2.64	**0.05**	1.14	0.72 - 1.81	0.57	1.69	0.75 - 3.82	0.20	1.22	0.71 - 2.09	0.47	1.04	0.62 - 1.76	0.87
LDH > ULN	Baseline	1.61	0.80 - 3.27	0.18	0.64	0.33 - 1.26	0.20	4.01	0.45 - 35.32	0.21	0.51	0.23 - 1.13	0.10	0.64	0.30 - 1.37	0.25
	Pre-LD	1.58	0.83 - 3.02	0.17	1.49	0.78 - 2.87	0.23	1.16	0.28 - 4.87	0.84	0.87	0.39 - 1.92	0.73	0.79	0.37 - 1.67	0.53
	Infusion	2.10	1.05 - 4.20	**0.04**	1.52	0.72 - 3.22	0.27	1.13	0.21 - 5.99	0.89	1.67	0.69 - 4.05	0.25	1.34	0.58 - 3.09	0.48
Ferritin*	Baseline	1.08	0.82 - 1.44	0.56	1.02	0.80 - 1.30	0.86	1.04	0.57 - 1.90	0.89	1.08	0.78 - 1.49	0.64	1.09	0.80 - 1.50	0.57
	Pre-LD	1.02	0.83 - 1.26	0.84	1.11	0.89 - 1.39	0.35	1.09	0.66 - 1.79	0.73	1.06	0.81 - 1.38	0.67	1.04	0.80 - 1.35	0.76
	Infusion	1.16	0.90 - 1.50	0.25	1.29	1.00 - 1.67	**0.05**	1.50	0.78 - 2.88	0.22	1.09	0.79 - 1.50	0.61	1.12	0.82 - 1.52	0.46

* Log2 transformed. CRP = C-reactive protein; CRS = cytokine release syndrome; CI = confidence interval; ICANS = immune effector cell-associated neurotoxicity syndrome; LDH = lactate dehydrogenase; OR = odds ratio; *p* = *p*-value; pre-LD = pre-lymphodepleting chemotherapy (day-15 until day -5); ULN = upper limit of normal. Bold indicates statistically significant values.

Table S5. Analysis of the association between patients' characteristics and CRS and ICANS

	CRS grade ≥ 2			ICANS grade ≥ 2			CRS grade ≥ 3			ICANS grade ≥ 3			CRS/ICANS grade ≥ 3		
	OR	95% CI	*p*	OR	95% CI	*p*	OR	95% CI	*p*	OR	95% CI	*p*	OR	95% CI	*p*
Age (> 65 vs ≤ 65)	0.91	0.45 - 1.85	0.80	1.54	0.35 - 6.72	0.57	1.68	0.83 - 3.41	0.15	1.41	0.61 - 3.25	0.42	1.67	0.77 - 3.65	0.20
Gender (male vs female)	0.77	0.39 - 1.50	0.44	0.51	0.12 - 2.11	0.35	0.42	0.21 - 0.83	**0.01**	0.44	0.20 - 0.96	**0.04**	0.41	0.20 - 0.88	**0.02**
Diagnosis (DLBCL vs rest)	1.38	0.73 - 2.62	0.32	1.25	0.65 - 2.40	0.49	NA	NA	-	0.70	0.32 - 1.55	0.38	1.11	0.53 - 2.33	0.78
ECOG (> 0 vs 0)	1.60	0.81 - 3.15	0.17	1.55	0.77 - 3.11	0.22	5.28	0.96 - 29.07	0.06	1.01	0.44 - 2.30	0.99	1.29	0.60 - 2.79	0.52
ECOG (> 1 vs ≤ 1)	1.10	0.31 - 3.83	0.88	1.90	0.54 - 6.65	0.31	6.66	1.08 - 41.05	**0.04**	2.12	0.58 - 7.76	0.25	2.99	0.84 - 10.66	0.09
Stage (III - IV vs I - II)	0.56	0.26 - 1.22	0.14	0.45	0.10 - 1.98	0.29	0.84	0.39 - 1.82	0.67	0.81	0.33 - 2.02	0.65	0.61	0.26 - 1.40	0.24
Bulky disease (yes vs no)	1.53	0.78 - 3.01	0.22	0.91	0.46 - 1.82	0.79	1.15	0.26 - 5.05	0.86	1.39	0.62 - 3.13	0.42	1.33	0.62 - 2.86	0.46
Extranodal sites (yes vs no)	1.11	0.56 - 2.19	0.77	0.91	0.46 - 1.81	0.79	0.86	0.20 - 3.81	0.84	1.43	0.60 - 3.39	0.41	1.18	0.53 - 2.59	0.69
Extranodal sites (≥ 2 vs < 2)	1.03	0.51 - 2.09	0.94	0.63	0.30 - 1.31	0.22	0.78	0.15 - 4.08	0.77	1.10	0.47 - 2.58	0.83	0.95	0.42 - 2.16	0.91
IPI score (> 1 vs ≤ 1)	0.90	0.35 - 2.33	0.83	3.15	1.03 - 9.67	**0.05**	1.22	0.13 - 11.83	0.86	1.64	0.47 - 5.70	0.43	1.42	0.45 - 4.46	0.55
IPI score (> 2 vs ≤ 2)	1.58	0.64 - 3.94	0.32	1.87	0.73 - 4.74	0.19	4.44	0.68 - 28.98	0.12	0.81	0.26 - 2.51	0.71	1.13	0.40 - 3.18	0.81

CI = confidence interval; CRS = cytokine release syndrome; DLBCL = Diffuse large B cell lymphoma; ECOG = eastern cooperative oncology group; ICANS = immune effector cell-associated neurotoxicity syndrome; IPI = international prognostic index; OR = odds ratio; *p* = *p*-value; Bold indicates statistically significant values.

Table S6. Univariable analyses of pre-LD (m-)EASIX using the different cut-offs proposed by the included studies

	CRS grade ≥ 2				ICANS grade ≥ 2				ICANS grade ≥ 3				CRS/ICANS grade ≥ 3			
	OR	95% CI	*p*	AUC	OR	95% CI	*p*	AUC	OR	95% CI	*p*	AUC	OR	95% CI	*p*	AUC
EASIX > 4.67[a]	-	-	-	-	-	-	-	-	-	-	-	-	0.61	0.16 - 2.27	0.46	0.52
EASIX > 2.1[a]	-	-	-	-	3.24	1.58 - 6.68	**< 0.01**	0.62	-	-	-	-	-	-	-	-
EASIX > 4.6[b]	1.81	0.64 - 5.07	0.26	0.53	-	-	-	-	-	-	-	-	-	-	-	-
m-EASIX ≥ 4[c]	-	-	-	-	-	-	-	-	1.28	0.55 - 2.99	0.56	0.53	-	-	-	-

[a]Proposed by Greenbaum et al. [8]; [b]Proposed by Korell et al. [17]; [c]Proposed by Acosta-Medina et al. [18]; AUC = area under the curve; CI = confidence interval; CRP = C-reactive protein; CRS = cytokine release syndrome; DLBCL = Diffuse large B cell lymphoma; EASIX = Endothelial Activation and Stress Index; m-EASIX = modified EASIX score; s-EASIX = simplified EASIX score; ICANS = immune effector cell-associated neurotoxicity syndrome; IPI = International Prognostic Index; IQR = interquartile range; LDH = lactate dehydrogenase; OR = odds ratio; *p* = *p*-value; pre-LD = pre-lymphodepleting chemotherapy (day-15 until day -5); ULN = upper limit of normal. Bold indicates statistically significant values.
